# Efficient low‐dose CT image enhancement using MobileMamba‐UNet with wavelet‐enhanced long‐range modeling

**DOI:** 10.1002/acm2.70680

**Published:** 2026-07-08

**Authors:** Jianfang Li, Haiyan Liu, Xiaoli Wang, Jianshu Hong

**Affiliations:** ^1^ School of Information Engineering Changsha Medical University Changsha China; ^2^ Guangzhou Institutes of Biomedicine and Health Chinese Academy of Sciences Guangzhou China; ^3^ Hunan Provincial Key Laboratory of the Traditional Chinese Medicine Agricultural Biogenomics, Hunan Provincial University Key Laboratory of the Fundamental and Clinical Research on Functional Nucleic Acid Changsha Medical University Changsha China; ^4^ Hunan Provincial Maternal and Child Health Care Hospital Changsha China; ^5^ China Spallation Neutron Source Dongguan China

**Keywords:** low‐dose CT, image denoising, Mamba, wavelet transform

## Abstract

**Background:**

Deep learning has become a dominant paradigm for low‐dose computed tomography (LDCT) image reconstruction. Nevertheless, existing approaches still struggle to simultaneously achieve accurate structural detail preservation and computational efficiency, particularly when handling long‐range contextual dependencies.

**Purpose:**

To design a lightweight yet effective LDCT reconstruction framework that captures both global contextual information and fine‐grained local details while maintaining low memory consumption and fast inference speed.

**Methods:**

We propose MobileMamba‐UNet, a hybrid neural network that integrates a MobileMamba backbone with a multi‐scale U‐Net architecture. The model incorporates a Wavelet Transform Enhanced Mamba mechanism to emphasize high frequency and diagnostically relevant structures, together with a multi‐receptive field feature interaction module that jointly models local textures and long‐range dependencies. All components are constructed with linear computational complexity to ensure efficiency in large‐scale LDCT reconstruction tasks.

**Results:**

Extensive experiments conducted on the Mayo‐2016 and Mayo‐2020 LDCT datasets demonstrate that MobileMamba‐UNet consistently outperforms existing CNN‐ and Transformer‐based methods. The proposed approach achieves superior image quality while significantly reducing memory usage and inference latency.

**Conclusions:**

MobileMamba‐UNet represents a promising approach for LDCT image reconstruction, balancing reconstruction performance with computational efficiency and practical applicability.

## 1 INTRODUCTION

Computed Tomography (CT) imaging plays a pivotal role in modern medical diagnostics, providing critical anatomical insights for disease detection, treatment planning, and therapy monitoring.^1^ However, the clinical utility of standard‐dose CT is constrained by the potential health risks associated with ionizing radiation. For instance, a single abdominal scan can expose a patient to a radiation dose equivalent to several years of natural background exposure.^2^ To mitigate these risks, Low‐Dose CT (LDCT) techniques have been widely adopted, utilizing strategies such as tube current modulation or sparse‐view acquisition. While these protocols significantly reduce radiation exposure, they inherently degrade image quality by introducing quantum noise and artifacts. This degradation obscures fine structural details, posing severe challenges for detecting subtle abnormalities such as early‐stage lung nodules or small liver lesions. Consequently, developing advanced denoising algorithms that balance radiation safety with high‐fidelity diagnostic performance has become a paramount research objective.

Early approaches to LDCT denoising relied primarily on analytical filtering^3^ and iterative reconstruction algorithms.^4^ The advent of deep learning has revolutionized this field, enabling powerful feature representations across diverse medical imaging tasks including patient privacy protection,^5^ survival prediction,^6^ multi‐view feature fusion,^7^, ^8^ and radiograph‐based disease classification.^9^ In LDCT denoising specifically, CNNs initially established the benchmark by deploying hierarchical layers to capture local texture information.^10^, ^11^ Subsequent innovations integrated multi‐scale learning strategies, such as U‐Net variants^12^, ^13^ and attention mechanisms,^14^ to further enhance structural retention. More recently, hybrid architectures that combine the local feature extraction of CNNs with the global modeling of Transformers have achieved strong results in LDCT denoising; for instance, HybridFormer^15^ integrates residual convolution blocks with Swin Transformer modules to jointly capture fine‐grained textures and long‐range contextual dependencies. Despite these advances, CNN‐based and hybrid methods still suffer from the restricted receptive field of convolution operations, limiting their ability to model the full range of anatomically correlated features such as vascular continuity and organ boundaries. Purely Transformer‐based methods further struggle with over‐smoothing at the cost of pixel‐level fidelity.

To address the limitations of local convolutions, Vision Transformers (ViTs) have emerged as a powerful alternative, leveraging self‐attention mechanisms to capture global context and long‐range dependencies. Pioneering models like TransCT^16^ and LITformer^17^ have demonstrated superior capability in differentiating noise from anatomical structures by modeling non local information. However, ViTs are hampered by a fundamental bottleneck: the computational complexity of the self‐attention mechanism scales quadratically with input size ($O(N^{2})$). This imposes prohibitive memory costs when processing high‐resolution 3D medical volumetric data; for example, processing a standard 512×512 CT slice often pushes modern GPUs to their memory limits. While optimization strategies such as windowed attention^18^ and token pruning have been proposed, they often compromise global context modeling, reintroducing local biases similar to CNNs. Thus, current methodologies face a critical trade‐off: maintaining global structural awareness at the cost of excessive computational demand, or simplifying computation at the risk of losing vital anatomical context.

Recently, the Mamba architecture,^19^, ^20^ based on State Space Models (SSMs), has garnered significant attention for its ability to model long sequences with linear complexity (O(N)), contrasting sharply with the quadratic cost of Transformers. Recent SSM‐based works such as VMamba^21^ and SegMamba^22^ have demonstrated that this efficiency translates effectively to high‐resolution medical image analysis, achieving competitive segmentation performance while significantly reducing memory consumption and inference latency compared to Transformer‐based counterparts. Among the evolving SSM variants, MobileMamba^23^ stands out as a particularly effective lightweight architecture whose unique characteristics directly address the bottlenecks of LDCT denoising: suppressing quantum noise while preserving subtle anatomical textures under strict computational constraints. However, the potential of adapting MobileMamba into a dense reconstruction framework like U‐Net for medical imaging remains unexplored.

To harness these advantages for medical diagnostics, we propose MobileMamba‐UNet, a novel low‐dose CT reconstruction network. Our architecture strategically integrates the MobileMamba backbone into a U‐shaped encoder‐decoder structure. By using MobileMamba as the core encoder, we exploit its Wavelet Transform Enhanced Mamba (WTE‐Mamba) module to specifically enhance the reconstruction of high‐frequency anatomical structures, including subtle tissue textures and small lesions, which are often overly smoothed by conventional CNNs or typical SSM architectures. Furthermore, we leverage the inherent Multi‐Receptive Field Feature Interaction (MRFFI) module within the MobileMamba blocks to capture a holistic feature representation, balancing long‐range global dependencies with local structural information. This integration allows MobileMamba‐UNet to achieve the high‐quality reconstruction typical of heavy Transformer models but with the inference speed and memory efficiency of lightweight networks. The primary contributions of this work are summarized as follows:

1. We present MobileMamba‐UNet, the first framework to integrate the state‐of‐the‐art MobileMamba architecture into a U‐Net‐based reconstruction pipeline. This novel synthesis effectively transfers the efficient long‐range modeling capabilities of MobileMamba to the domain of LDCT denoising.
2. We demonstrate that the specific components of MobileMamba block are intrinsically suited for solving the texture‐loss and noise‐artifact trade‐off in medical imaging. Our work validates that leveraging these frequency‐aware and multi‐scale mechanisms significantly improves the preservation of diagnostic details compared to standard backbone choices.
3. Through comprehensive validation on multiple public datasets, we demonstrate that MobileMamba‐UNet outperforms state‐of‐the‐art Transformer and CNN baselines in both quantitative metrics and visual quality, while requiring significantly lower computational resources, thereby enhancing its suitability for real‐world deployment.

## 2 METHODS

### 2.1 Architecture overview

MobileMamba‐UNet is specifically designed to efficiently reconstruct LDCT images while preserving both local textural details and global anatomical context. The architecture integrates the MobileMamba framework,^23^ which operates with linear computational complexity, into the hierarchical multi‐scale structure of U‐Net, providing a strong foundation for LDCT reconstruction. The proposed model follows an encoder–decoder paradigm that combines global context modeling with a rich set of local feature extraction mechanisms.

The core design philosophy of MobileMamba‐UNet is to overcome the limitations of existing methods by exploiting the complementary strengths of SSMs and convolutional operations. By incorporating MobileMamba's efficient sequence modeling within a multi‐scale hierarchical framework, the proposed architecture achieves lower computational cost compared to Transformer‐based alternatives while retaining a global receptive field, which is essential for preserving complete anatomical structures. The encoder‐decoder design supports progressive feature abstraction and reconstruction, enabling effective noise suppression and retention of diagnostically relevant structural details across multiple spatial scales.

### 2.2 Architectural components

**FIGURE 1:**
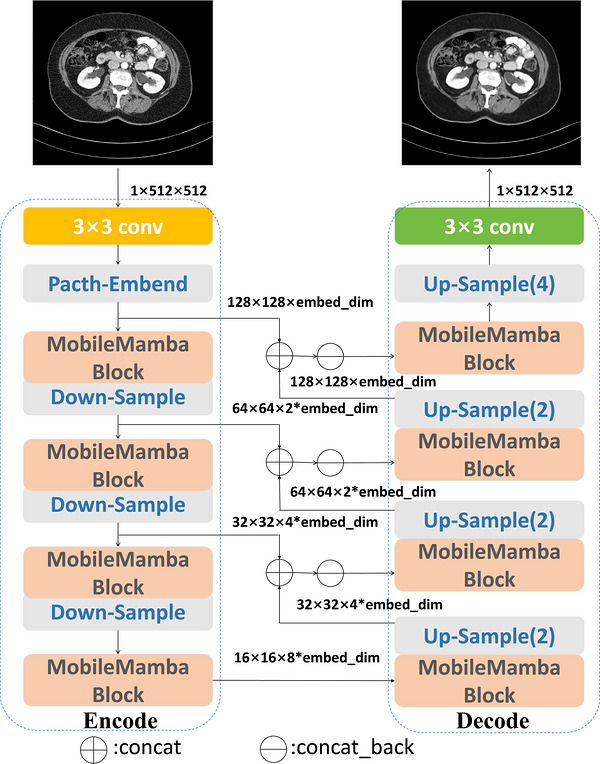
The overall architecture of MobileMamba‐UNet.

The proposed architecture, illustrated in Figure 1, consists of three main components.

**Encoder**: A four‐stage MobileMamba backbone that progressively extracts hierarchical features while remaining computationally efficient. At each stage, strided convolutions with stride 2 perform downsampling, reducing the spatial resolution from H×W to H/8×W/8 while expanding the channel dimension from C – 4C. This design supports effective multi‐scale feature learning and captures long‐range dependencies with linear complexity O(n), avoiding the quadratic cost $O(n^{2})$ associated with ViT‐based methods.

**Decoder**: A symmetric upsampling pathway that reconstructs high‐resolution features via gated bidirectional skip connections. Transposed convolutions with stride 2 are applied at each stage to restore feature maps while reducing the channel dimensions. These skip connections adaptively combine multi‐scale features from corresponding encoder stages, preserving both local spatial details and global contextual information. A final 3×3 convolution with instance normalization and ReLU activation is applied to generate the output, effectively suppressing noise while retaining structural information.

**MRFFI Module**: A hybrid processing module that combines the Wavelet Transform Enhanced Mamba (WTE‐Mamba) and Multi‐Kernel Depthwise Convolution (MK‐DeConv) mechanisms to jointly capture global contextual dependencies and local structural details.

Given an input LDCT image $X\in R^{H\times W}$, the network applies a multi‐resolution processing strategy across hierarchical spatial scales. The encoder extracts progressively abstract multi‐scale representations, while the decoder reconstructs high‐quality feature maps. The bidirectional skip connections facilitate information exchange between the two pathways, preserving both global context and local structural nuances, thereby ensuring high‐fidelity reconstruction.

### 2.3 PatchEmbed

The PatchEmbed module serves as the initial feature extraction stage in MobileMamba‐UNet, transforming input LDCT images into compact feature representations. It consists of three consecutive ConvBNReLU layers, each employing a 16×16 convolution with stride 2. Within each layer, the convolution captures features over a large receptive field, followed by batch normalization for training stability and ReLU activation for nonlinearity.

With stride 2 applied at each layer, the spatial resolution is progressively reduced while feature abstraction increases. After three layers, the spatial resolution is reduced by a factor of 8, and the channel dimension is correspondingly expanded. This design enables effective multi‐scale feature extraction and provides a smooth transition from low‐level image information to high‐level representations for subsequent processing by the MobileMamba blocks.

### 2.4 MobileMamba block

**FIGURE 2:**
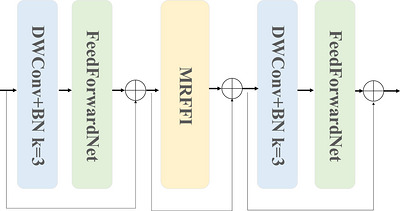
Detailed architecture of the MobileMamba Block.

The MobileMamba Block is the core computational unit of the proposed architecture, designed to integrate local feature extraction with global context modeling. As shown in Figure 2, the block adopts a residual structure consisting of three sequential sub‐modules: two DWConv‐FFN sub‐blocks sandwiching a central MRFFI module. Each sub‐block is equipped with an independent residual connection to ensure efficient gradient flow and hierarchical feature learning. Specifically, the input features are processed by a “DWConv + BN→FFN” sequence twice, with the MRFFI operation performed in between.

In each DWConv‐FFN sub‐block, the input features first pass through depthwise convolution followed by batch normalization, and the resulting features are then processed by a Feed‐Forward Network (FFN). This DWConv→FFN sequence is applied twice within the MobileMamba Block. The depthwise convolution uses a 3×3 kernel with batch normalization, which efficiently extracts spatially localized features with linear computational complexity relative to input size, making the architecture well‐suited for resource‐constrained deployment.

The FFN serves as the feature projection component, employing a two‐layer structure to transform feature representations. Each FFN consists of: (1) a linear projection layer that expands the input dimension from C –4C, followed by GELU activation; and (2) a subsequent projection layer that restores the dimension from 4C back to C. This expansion‐compression strategy allows the network to learn richer representations in the expanded latent space while maintaining dimensional consistency. The expansion ratio of 4 follows established practices in Transformer architectures, balancing representational capacity with computational efficiency. Layer normalization is applied before each FFN to stabilize training.

A key innovation in the MobileMamba Block is the integration of the MRFFI module at its center, positioned between the two DWConv‐FFN sub‐blocks. The MRFFI module enables simultaneous modeling of short‐range spatial dependencies through convolution and long‐range contextual dependencies through SSM‐based mechanisms. By capturing multi‐scale contextual information, the module preserves fine textural details while maintaining global anatomical structural coherence.

### 2.5 MRFFI module

**FIGURE 3:**
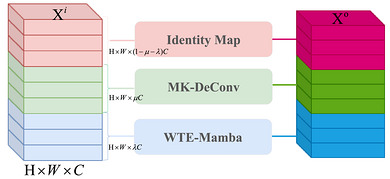
Illustration of the MRFFI module.

To capture both global contextual dependencies and local textural characteristics, we propose the MRFFI module. As illustrated in Figure 3, given an input feature tensor $X^{i}\in R^{H\times W\times C}$, the module processes features through three parallel branches and fuses their outputs to produce a refined representation $X^{o}$.

The input $X^{i}$ is split across three branches: an Identity Map branch, the WTE‐Mamba branch, and the MK‐DeConv branch. To balance global and local feature extraction, we adopt a depth‐dependent channel allocation strategy. Specifically, the channel ratios for the Identity Map (μ) and WTE‐Mamba (λ) are set to {0.8,0.7,0.6,0.5} and {0.2,0.2,0.3,0.4}, respectively, across the four network stages. This design allows shallow layers to focus more on preserving local details (larger μ), while deeper layers progressively shift toward global context modeling (larger λ).

The Identity Map branch maintains a direct pathway to preserve original local details and fine‐grained textures, preventing the over‐smoothing effects that often arise from large‐receptive‐field operations and ensuring that high‐frequency information is retained. The WTE‐Mamba branch leverages selective attention mechanisms to capture long‐range spatial correlations efficiently, providing the global semantic cues necessary for understanding complex anatomical structures in LDCT reconstruction. The MK‐DeConv branch employs multi‐kernel depthwise convolutions to aggregate features at multiple scales, refining spatial details and recovering fine structures that may be degraded during downsampling. Finally, the outputs from all three branches are concatenated along the channel dimension, synergistically integrating global context with local spatial precision and producing enriched representations that capture both large‐scale anatomical morphology and subtle texture patterns.

#### 2.5.1 Efficient multi‐kernel depthwise convolution

To enhance the extraction of local and multi‐scale information in LDCT reconstruction, we design the MK‐DeConv module within the MRFFI framework. This module complements the global modeling capability of WTE‐Mamba by focusing on spatial features across different receptive fields.

**FIGURE 4:**
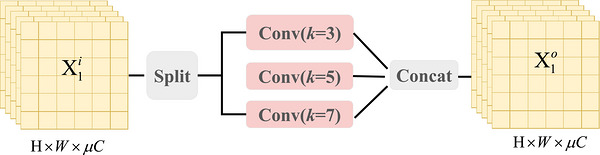
Architecture of the MK‐DeConv module.

As illustrated in Figure 4, the input feature $X_{1}^{i}\in R^{H\times W\times \mu C}$ is evenly divided along the channel dimension into three groups. Each group is independently processed by a depthwise convolution with a different kernel size, namely k=3, k=5, and k=7, to capture local, intermediate, and broader contextual information, respectively. The resulting multi‐scale features are then concatenated along the channel dimension to produce the output $X_{1}^{o}\in R^{H\times W\times \mu C}$. This multi‐kernel design enriches receptive field diversity and enhances the model's ability to integrate spatial information at multiple scales.

#### 2.5.2 Wavelet transform enhanced Mamba

**FIGURE 5:**
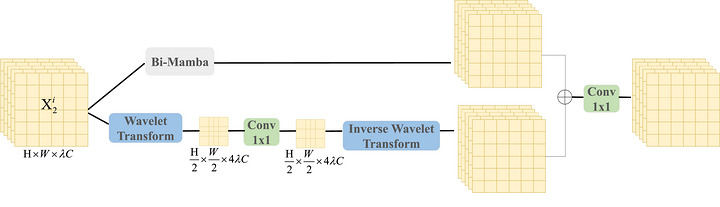
Architecture of the WTE‐Mamba module.

To bridge global context modeling with high‐frequency detail preservation, we introduce the Wavelet Transform Enhanced Mamba (WTE‐Mamba) block as one of the three parallel branches within the MRFFI module. As illustrated in Figure 5, the input features $X_{G}^{i}\in R^{H\times W\times \xi C}$ are processed through two parallel pathways: (1) a Bidirectional Mamba (Bi‐Mamba) pathway that applies linear projection, convolution, and bidirectional SSM scanning to capture long‐range dependencies in both forward and backward directions; and (2) a Wavelet pathway that performs discrete wavelet transform (WT) to decompose features into multifrequency sub‐bands ($f_{LL}$, $f_{LH}$, $f_{HL}$, and $f_{HH}$), applies convolution to each sub‐band, and reconstructs spatial resolution via inverse wavelet transform (IWT). The outputs from both pathways are fused through element‐wise addition to yield the final output $X_{G}^{O}$.

The original Mamba block is designed to process one‐dimensional sequences in a single direction. To better capture feature representations from multiple directions, we design a Bidirectional Mamba (Bi‐Mamba) structure. The selected channel subset $X_{G}^{i}\in R^{H\times W\times \lambda C}$ is processed by a Mamba module that applies linear projection, convolution, and bidirectional SSM scanning to learn global contextual dependencies from both forward and backward directions. This process is formulated as Equation (1):

(1) $$\begin{array}{cc} x_{m1}^{I} & =\mathrm{SSM}\left(\sigma \left(\mathrm{Conv}\left(\mathrm{Linear}\left(X_{G}^{i}\right)\right)\left[:,\lambda C\right]\right)\right),\\ x_{m2}^{I} & =\sigma \left(\mathrm{Linear}\left(X_{G}^{i}\right)\left[\lambda C:\right]\right),\\ X_{m}^{O} & =\mathrm{Linear}\left(x_{m1}^{I}⊗x_{m2}^{I}\right). \end{array}$$

Here, $x_{m1}^{I}$ is the output of the SSM applied to the first λC channels after linear projection, convolution, and activation, capturing long‐range dependencies through bidirectional scanning; $x_{m2}^{I}$ is the activated linear transformation of the remaining channels, serving as a gating signal; and $X_{m}^{O}$ is the final Mamba output obtained by element‐wise multiplication (⊗) of the two paths followed by a linear projection.

Concurrently, $X_{G}^{i}$ undergoes WT to decompose features into multifrequency components, producing one low‐frequency sub‐band and three high‐frequency sub‐bands. The low‐frequency sub‐band retains the primary structural information, while the high‐frequency sub‐bands capture edge and texture details. These sub‐bands are convolved individually and recombined via IWT to restore spatial resolution, yielding $X_{w}^{O}\in R^{H\times W\times \lambda C}$. The process is formulated as Equation (2):

(2) $$\begin{array}{cc} X_{wt}^{I} & =\mathrm{WT}\;\left(X_{G}^{i}\right),\\ X_{w}^{O} & =\mathrm{IWT}\;\left(\mathrm{Conv}\;\left(X_{wt}^{I}\right),\;\left[f_{LL},\;f_{LH},\;f_{HL},\;f_{HH}\right]\right),\\ X_{G}^{O} & =X_{m}^{O}+X_{w}^{O}, \end{array}$$

where, $X_{wt}^{I}$ denotes the wavelet‐domain feature representation containing four sub‐bands: $f_{LL}$ (low‐frequency components preserving primary structural information), $f_{LH}$ (horizontal high‐frequency components capturing horizontal edges), $f_{HL}$ (vertical high‐frequency components capturing vertical edges), and $f_{HH}$ (diagonal high‐frequency components capturing diagonal edges and texture details). After the WT, convolution is applied to each sub‐band, which benefits from a larger effective receptive field and lower computational cost compared to operations at the original scale. The IWT then reconstructs spatial resolution by synthesizing all processed frequency components. The fused output $X_{G}^{O}$ combines global context from the Mamba pathway and multifrequency local information from the wavelet pathway via element‐wise addition.

This dual‐pathway fusion allows the WTE‐Mamba branch to simultaneously leverage long‐range dependencies from the SSM structure and retain fine‐grained high‐frequency textures from the wavelet‐based path. In the context of LDCT reconstruction, this is particularly beneficial: the SSM component helps restore anatomical coherence and large‐scale structural consistency, while the wavelet component ensures that subtle edge features, texture cues, and high‐frequency patterns are preserved, all of which are critical for diagnostic quality. By allocating only a fraction λ of channels to this branch, the model controls computational overhead while enriching representations with multi‐scale and frequency‐domain information. In practice, gradually increasing λ with network depth further enhances the ability to model global structures in later stages, while earlier layers remain focused on local detail extraction. This design effectively complements the MK‐DeConv and Identity branches within the MRFFI module, producing a balanced feature interaction mechanism that excels in both local spatial sensitivity and global context integration.

## 3 EXPERIMENTS

### 3.1 Dataset

We conducted a comprehensive evaluation of our proposed method on two publicly accessible datasets: the 2016 AAPM‐Mayo Clinic Low Dose CT Grand Challenge dataset (Mayo‐2016)^24^ and the Low Dose CT Image and Projection Data collection (Mayo‐2020).^25^ All CT volumes were reconstructed into slices of 512×512 pixels with a thickness of 3.0 mm.

The Mayo‐2016 dataset contains abdominal CT scans from 10 patients, each comprising paired NDCT and LDCT images. For our experiments, we adhered to the standard subject‐independent evaluation protocol, allocating data from nine patients (2167 image pairs) for model training and the complete scan data of one patient (L506, 211 pairs) for testing.

To further assess the model's generalization capabilities across different anatomical regions and dose levels, we utilized the Mayo‐2020 dataset. This collection features scans of the chest and liver. Specifically, our experiments utilized chest scans acquired at 10% of the normal radiation dose and liver scans at 25% dose. From this dataset, we partitioned the data into a training set of 4068 image pairs (2464 chest, 1604 liver) and a testing set of 517 pairs (303 chest, 214 liver), ensuring no patient overlap between the two sets.

### 3.2 Implementation details

We implemented MobileMamba‐UNet in PyTorch and trained the model on four NVIDIA V100 GPUs with 32 GB memory each. The network was optimized using the Adam optimizer with an initial learning rate of $1\times 10^{-4}$ and a weight decay of $1\times 10^{-4}$. We adopted a cosine annealing scheduler with a minimum learning rate of $1\times 10^{-6}$ over a total of 200 epochs. Training was conducted for 200 epochs with a batch size of 16, using randomly cropped patches of size 256×256 from the original 512×512 slices. To ensure boundary consistency during patch‐based reconstruction, MobileMamba‐UNet leverages its inherent long‐range modeling capability. The MRFFI module effectively captures global contextual information, maintaining feature coherence across patch transitions and mitigating potential boundary artifacts. During inference, the model processes full 512×512 slices directly without any sliding‐window strategy, which is feasible due to the linear computational complexity of the architecture.

Our data augmentation strategy included rotations by $90^{\circ }$, $180^{\circ }$, and $270^{\circ }$, horizontal flipping with a probability of 0.5, and random intensity shifts (±10%) to enhance model generalization. These axis‐aligned rotations preserve CT image fidelity by maintaining the original grid structure and Hounsfield unit (HU) values without interpolation‐induced artifacts, which is critical for diagnostic accuracy in medical imaging. The loss function combined L1 loss (weighted at 0.8) and SSIM loss (weighted at 0.2) to balance pixel‐wise fidelity and structural integrity. We configured MobileMamba‐UNet with 2, 2, 2, 2 MobileMamba Blocks across the four network stages, progressively increasing feature dimensions from 64 to 512 channels through the encoder path. The expansion ratio in FeedForwardNet modules was set to 4.0, and we employed multi‐kernel configurations 3, 5, 7 in the MK‐DeConv module to capture features at different spatial scales. The implementation code of MobileMamba‐UNet will be publicly available at https://github.com/hhhljf/MobileMamba‐UNet.

### 3.3 Evaluation metrics

We comprehensively evaluated reconstruction quality using five complementary metrics that assess different aspects of image fidelity. Let I denote the reconstructed image and K denote the ground‐truth reference image, both of size H×W.

- **Peak Signal‐to‐Noise Ratio (PSNR)** measures pixel‐wise accuracy on a logarithmic scale, with higher values indicating better reconstruction quality. It is defined as Equation 3:
(3) $$\begin{array}{c} \text{PSNR}\left(I,K\right)=10\cdot \log_{10}\left(\frac{{\text{MAX}}_{I}^{2}}{\text{MSE}(I,K)}\right), \end{array}$$
  where, ${\text{MAX}}_{I}$ denotes the maximum possible pixel value of the image (e.g., 1.0 for normalized images or 255 for 8‐bit images), and MSE(I,K) represents the Mean Squared Error between the reconstructed image I and the ground‐truth image K.
- **Structural Similarity Index (SSIM)** evaluates perceptual quality by comparing luminance, contrast, and structural information between reconstructed and reference images. It is defined for two image windows x and y as Equation 4:
(4) $$\begin{array}{c} \text{SSIM}\left(x,y\right)=\frac{\left(2\mu_{x}\mu_{y}+c_{1}\right)\left(2\sigma_{xy}+c_{2}\right)}{\left(\mu_{x}^{2}+\mu_{y}^{2}+c_{1}\right)\left(\sigma_{x}^{2}+\sigma_{y}^{2}+c_{2}\right)}, \end{array}$$
  where, $\mu_{x}$ and $\mu_{y}$ are the local means, $\sigma_{x}^{2}$ and $\sigma_{y}^{2}$ are the local variances, $\sigma_{xy}$ is the cross‐covariance, and $c_{1},c_{2}$ are small constants to stabilize the division. The final SSIM score is the mean of the SSIM values computed over all local windows.
- **Root Mean Squared Error (RMSE)** quantifies the average magnitude of reconstruction errors in the original intensity scale. Lower values are better. It is defined as the square root of the Mean Squared Error (MSE), as shown in Equation 5:
(5) $$\begin{array}{c} \text{RMSE}\left(I,K\right)=\sqrt{\frac{1}{HW}\sum_{i=1}^{H}\sum_{j=1}^{W}{\left[I\left(i,j\right)-K\left(i,j\right)\right]}^{2}}, \end{array}$$
- **Learned Perceptual Image Patch Similarity (LPIPS)** measures the perceptual difference between two images using features extracted from a pretrained deep neural network(ReNet‐50). Lower values indicate higher perceptual similarity. The distance is computed as Equation 6:
(6) $$\begin{array}{c} d\left(I,K\right)=\sum_{l}\frac{1}{H_{l}W_{l}}\sum_{h,w}\left|\right|w_{l}⊙\left(f_{hw}^{l}-{\hat{f}}_{hw}^{l}\right){\left|\right|}_{2}^{2}, \end{array}$$
  where, $f_{hw}^{l}$ and ${\hat{f}}_{hw}^{l}$ are the feature activations for images I and K at spatial location (h,w) in layer l of the network, and $w_{l}$ are learnable channel‐wise weights.
- **Visual Information Fidelity (VIF)** assesses image quality based on information theory, quantifying the amount of information shared between the reference and distorted images from the perspective of the human visual system. Higher values are better. It is defined as the ratio of two mutual informations, as shown in Equation 7:
(7) $$\begin{array}{c} \text{VIF}\left(I,K\right)=\frac{\sum_{s\in S}I\left(C^{s};F^{s}|M^{s}\right)}{\sum_{s\in S}I\left(C^{s};E^{s}|M^{s}\right)}, \end{array}$$
  where, I(·;·|·) is the conditional mutual information, $C^{s}$ is the random field from the reference image in a specific sub‐band s, while $E^{s}$ and $F^{s}$ are the corresponding random fields for the reference and test images after passing through the HVS model. $M^{s}$ models the visual masking effect.

For all experiments, metrics were computed on the test set using the full [‐1000, 2000] HU range to ensure a comprehensive evaluation, unless otherwise specified.

### 3.4 Ablation studies

To validate the effectiveness of the core components of our proposed MobileMamba‐UNet, we conducted a series of ablation studies on the Mayo‐2016 dataset. Starting from a baseline model, we incrementally added each proposed module and evaluated the performance at each step to assess their individual contributions.

#### 3.4.1 Impact of WT in WTE‐Mamba

To optimize the WT component within the WTE‐Mamba module, we compared different wavelet families and decomposition levels, as shown in Table 1. Among the tested wavelet families, including Haar, Daubechies‐4, Coiflets‐2, Symlets‐4, and Biorthogonal‐2.2, the biorthogonal wavelet achieves the best performance with a PSNR of 40.31 dB and an SSIM of 0.881. This superior performance can be attributed to the perfect reconstruction property of biorthogonal wavelets, as well as their asymmetric analysis and synthesis filters, which are better suited to preserving both edge information and smooth regions in medical images.

**TABLE 1:** Performance comparison with different wavelet configurations in WTE‐Mamba module. The metrics are computed within the range of [‐1000, 2000] HU. Best results are in **bold**.

Wavelet Type	Levels	PSNR (dB)	SSIM
Haar	2	39.62	0.818
Daubechies‐4	2	39.65	0.822
Coiflets‐2	2	39.73	0.828
Symlets‐4	2	39.86	0.838
Biorthogonal‐2.2	2	**40.31**	**0.881**

#### 3.4.2 Effectiveness of proposed modules

We investigated the individual contributions of WTE‐Mamba part and MK‐DeConv part in MRFFI modules. As shown in Table 2, our baseline model, a standard U‐Net with Mamba blocks, achieves a PSNR of 39.21 dB. Integrating the MRFFI module improves performance by enhancing multi‐scale feature fusion, boosting PSNR to 39.75 dB. Further incorporating the WTE‐Mamba module, which leverages WTs for better feature representation, yields the most significant improvement, reaching our final PSNR of 40.31 dB. This demonstrates that both proposed modules are critical to the model's overall performance.

**TABLE 2:** Ablation study on the effectiveness of the WTE‐Mamba and MRFFI modules. Best results are in **bold**.

Baseline	+MK‐DeConv	+WTE‐Mamba	PSNR(dB)
✓			39.21
✓	✓		39.75
✓	✓	✓	**40.31**

#### 3.4.3 Analysis of loss function components

To justify our choice of a combined loss function, we evaluated the model's performance using only L1 loss, only SSIM loss, and the weighted combination (0.8∗L1+0.2∗SSIM). Table 3 shows that using L1 loss alone achieves a high PSNR but a lower SSIM, indicating good pixel‐wise accuracy but suboptimal structural preservation. Conversely, SSIM loss alone improves structural similarity but at the cost of pixel‐level fidelity. Our combined loss function achieves the best balance, outperforming the individual components across both metrics. This confirms that integrating both pixel‐based and perceptual losses is essential for high‐quality image reconstruction.

**TABLE 3:** Ablation study on the components of the loss function. Best results are in **bold**.

Loss Function	PSNR (dB)	SSIM
L1 Loss only	39.85	0.872
SSIM Loss only	39.42	0.879
L1 + SSIM	**40.31**	**0.881**

#### 3.4.4 Analysis of PatchEmbed design

To validate the design choice of using three consecutive stride‐2 convolutions in the PatchEmbed module, we conducted an ablation study comparing our proposed configuration against a standard ViT‐style single‐projection embedding, which directly maps each nonverlapping 16×16 patch to a token via a single convolutional layer with kernel size 16 and stride 16. As shown in Table 4, replacing the three‐stride convolution stack with a single‐projection embedding leads to a noticeable degradation in reconstruction quality, with PSNR dropping by 0.18 dB and SSIM decreasing by 0.002. This performance gap can be attributed to the fact that progressive downsampling introduces additional nonlinearities between stages, enabling the network to learn richer low‐level representations before tokens are formed, and that the gradual spatial compression better retains fine‐grained edge and texture information that is diagnostically relevant in LDCT images. These results confirm that the three‐stride PatchEmbed design is a meaningful architectural choice that contributes measurably to the overall reconstruction performance of MobileMamba‐UNet.

**TABLE 4:** Ablation study on PatchEmbed design evaluated on the Mayo‐2016 test set. Best results are in **bold**.

PatchEmbed design	PSNR (dB)	SSIM
Single projection (ViT‐style)	40.13	0.878
Three‐stride convolutions (Ours)	**40.31**	**0.881**

### 3.5 Comparison with state‐of‐the‐art methods

To comprehensively evaluate the effectiveness of MobileMamba‐UNet, we compared it against several representative state‐of‐the‐art LDCT denoising methods, covering three categories of approaches. The first category consists of early CNN‐based methods, namely RED‐CNN^26^ and EDCNN.^27^ The second category includes advanced Transformer‐based models, specifically CTformer^28^ and Swin‐Unet.^29^ The third category features the recent Mamba‐based method CT‐Mamba.^30^ Both quantitative and qualitative evaluations were conducted, along with an analysis of model efficiency in terms of parameter count and the trade‐off between computational complexity and reconstruction performance.

#### 3.5.1 Performance on Mayo‐2016 dataset

Our primary quantitative evaluation was carried out on the standard Mayo‐2016 benchmark dataset. As summarized in Table 5, when evaluated over the full HU range of [‐160, 240], MobileMamba‐UNet consistently achieves the best overall performance among all competing methods. Compared to CT‐Mamba, the most relevant Mamba‐based baseline, our model not only improves PSNR by 0.08 dB and reduces perceptual error (LPIPS) by 21%, but also achieves this with only one‐fourth of the parameters. This demonstrates that the proposed wavelet‐enhanced hybrid design effectively enhances both pixel‐level fidelity and perceptual quality while maintaining lightweight efficiency. These consistent improvements highlight the strong generalization capability of MobileMamba‐UNet for LDCT noise suppression and anatomical detail preservation within the soft‐tissue window. To assess whether the observed improvements are statistically significant, we performed paired two‐sided t‐tests on per‐slice PSNR and SSIM scores across all test slices, comparing MobileMamba‐UNet against each baseline. As reported in Table 5, all comparisons yield p<0.05, confirming that the performance gains of our method are statistically significant.

**TABLE 5:** Quantitative comparison on the **Mayo‐2016** dataset within the soft tissue **[‐160, 240] HU** window. The test dataset is L506. Results are reported as mean±std across test slices. p‐values are from paired two‐sided t‐tests on per‐slice PSNR between each method and Ours. Best results are in **bold**, second best are underlined.

Method	PSNR (dB) ↑	SSIM ↑	RMSE ↓	LPIPS ↓	VIF ↑	Params(M)	*p*‐value
LDCT	29.249±1.823	0.8759±0.0312	14.242±2.156	0.158	0.861	—	<0.001
RED‐CNN	32.922±1.247	0.9074±0.0198	9.217±1.334	0.059	0.913	1.856	<0.001
EDCNN	32.923±1.251	0.9069±0.0201	9.208±1.329	0.061	0.912	0.081	<0.001
CTformer	33.124±1.189	0.9125±0.0187	9.008±1.287	0.052	0.919	$\underline{1.451}$	<0.001
Swin‐Unet	33.365±1.152	0.9148±0.0176	8.774±1.198	0.045	0.924	29.10	<0.001
CT‐Mamba	$\underline{33.502}\pm \underline{1.098}$	$\underline{0.9179}\pm \underline{0.0162}$	$\underline{8.314}\pm \underline{1.143}$	$\underline{0.038}$	$\underline{0.954}$	21.28	0.012
**Ours**	33.582±1.071	0.9189±0.0158	8.053±1.112	0.030	0.989	5.71	—

**FIGURE 6:**
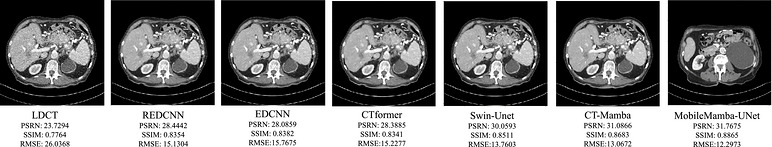
Qualitative comparison of different LDCT denoising methods on a challenging abdominal CT slice from the test set. All images are displayed with a window of [‐160, 240] HU. The proposed MobileMamba‐UNet produces cleaner and sharper results, with clearer anatomical structures and reduced noise artifacts compared to other methods.

To further demonstrate the quantitative and qualitative superiority of our MobileMamba‐UNet, we select a challenging abdominal CT slice from the test set for detailed comparison. Figure 6 presents denoised results obtained by different SOTA methods, along with their corresponding quantitative metrics. As visually evident, our model produces images with clearer structural boundaries, higher contrast, and more faithfully preserved anatomical details, while effectively suppressing residual noise and artifacts.

**FIGURE 7:**
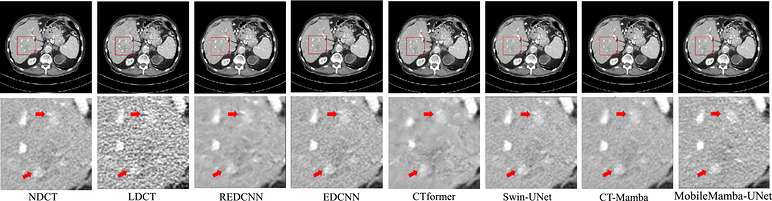
Detailed visual comparison on a randomly selected abdominal CT slice from the test set (displayed in the window of [‐160, 240] HU). Our MobileMamba‐UNet achieves better noise suppression while preserving fine details and tissue boundaries, producing results closest to the NDCT reference.

Figure 7 provides a detailed visual comparison. It shows complete abdominal CT images in the top row and zoomed‐in areas in the bottom row. The figure compares the normal‐dose CT images (which serve as the ideal reference) with the noisy low‐dose CT input images and the results from different denoising methods. The red arrows highlight areas where our MobileMamba‐UNet successfully restores fine details that were hidden by noise in the original low‐dose images. Compared to other methods, our approach better balances two important goals: removing noise while keeping important tissue edges and small anatomical structures intact. As a result, our method produces images that are closest to the normal‐dose reference images.

#### 3.5.2 Performance on Mayo‐2020 dataset

To further validate the generalization performance of our proposed model across different anatomical structures and dose levels, we conducted experiments on the Mayo‐2020 dataset, which includes chest scans acquired at 10% of the normal radiation dose and liver scans at 25%. All CT volumes were reconstructed into 512×512 slices within a display range of [‐1000, 2000]HU. The quantitative results are summarized in Table 6. Our method achieved the best performance across all evaluated metrics, surpassing the state‐of‐the‐art CT‐Mamba model by a notable margin. Compared to traditional CNN‐based approaches such as RED‐CNN and EDCNN, our model demonstrates significant improvements in both quantitative accuracy and perceptual quality, particularly under severe noise conditions. While transformer‐based architectures like CTformer and Swin‐UNet improved global feature modeling, they still exhibited degradation in subtle tissue boundaries and texture consistency. In contrast, our MobileMamba‐UNet effectively integrates multi‐scale spatial dependencies with state‐space representations, enabling robust denoising performance across diverse anatomical regions and dose levels. Paired two‐sided t‐tests on per‐slice PSNR scores confirm that all improvements over baseline methods are statistically significant (p<0.05), as reported in Table 6.

**TABLE 6:** Performance comparison with state‐of‐the‐art LDCT denoising methods on the **Mayo‐2020** dataset. The metrics are computed within the range of [‐1000, 2000] HU. Results are reported as mean±std across test slices. p‐values are from paired two‐sided t‐tests on per‐slice PSNR between each method and Ours. Best results are in **bold**, second best are underlined.

Method	PSNR (dB) ↑	SSIM ↑	RMSE($10^{-2}$) ↓	LPIPS ↓	VIF ↑	*p*‐value
LDCT	32.821±2.341	0.7121±0.0423	1.018±0.187	0.215	0.654	<0.001
RED‐CNN	36.403±1.876	0.7812±0.0358	0.662±0.134	0.112	0.813	<0.001
EDCNN	39.482±1.543	0.8742±0.0281	0.421±0.098	0.071	0.889	<0.001
CTformer	36.367±1.912	0.8101±0.0341	0.538±0.118	0.105	0.825	<0.001
Swin‐Unet	39.715±1.498	0.8622±0.0267	0.381±0.089	0.063	0.901	<0.001
CT‐Mamba	$\underline{40.222}\pm \underline{1.387}$	$\underline{0.8903}\pm \underline{0.0241}$	$\underline{0.362}\pm \underline{0.081}$	$\underline{0.052}$	$\underline{1.065}$	0.018
**Ours**	40.328±1.356	0.8918±0.0235	0.322±0.076	0.0485	1.112	—

**FIGURE 8:**
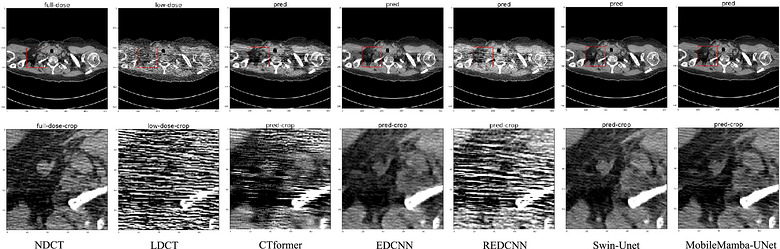
A detailed visual comparison on an abdominal CT slice randomly selected from the test set of the Mayo‐2020, displayed within the intensity window of [−1000,2000] HU.

Figure 8 shows a detailed comparison of how well different methods remove noise. The top row shows complete cross‐sectional abdominal CT images, and the bottom row shows close‐up views of the areas marked with red boxes to better see small anatomical details. In these close‐up areas, our MobileMamba‐UNet creates sharper tissue edges and preserves fine structures better while removing noise effectively. Other methods either still show visible noise or make important features blurry, but MobileMamba‐UNet produces results that look most similar to the normal‐dose CT reference images. Our method achieves a better balance between removing noise and keeping anatomical details clear.

#### 3.5.3 Noise power spectrum (NPS) analysis

To further evaluate the perceptual realism of the reconstructed images beyond pixel‐level metrics, we conducted NPS analysis on a representative subset of the Mayo‐2016 test cases. The 2D NPS was estimated from 64×64 ROIs selected from homogeneous background regions of each test slice, and computed as:

(8) $$\text{NPS}\left(f_{x},f_{y}\right)=\frac{\Delta x\cdot \Delta y}{N_{x}\cdot N_{y}}{\left|F\left[I\left(x,y\right)-\hat{I}\left(x,y\right)\right]\right|}^{2},$$

where, F denotes the 2D discrete Fourier transform, I(x,y) is the pixel intensity within the ROI, $\hat{I}\left(x,y\right)$ is a second‐order polynomial fit used to remove low‐frequency trends, and Δx, Δy are the pixel spacings. The 1D radial NPS profile was then obtained by azimuthal averaging and further averaged across all ROIs and test slices. As shown in Table 7, CNN‐based methods tend to over‐suppress high‐frequency components, shifting $f_{\text{peak}}$ toward lower frequencies and yielding NPS AUC values below the NDCT reference, which is indicative of over‐smoothing, while Transformer‐based methods show a more balanced but still notable deviation. MobileMamba‐UNet achieves the closest match to the NDCT reference across all three NPS metrics, with $f_{\text{peak}}$ and AUC deviating by less than 3% from the NDCT target, confirming that the proposed method better preserves realistic noise texture while effectively suppressing excessive noise.

**TABLE 7:** Quantitative NPS analysis on the Mayo‐2016 test set. $f_{\text{peak}}$ denotes the spatial frequency at peak NPS magnitude. AUC is the normalized area under the 1D radial NPS curve. NDCT serves as the reference target. Best results among denoising methods are in **bold**.

Method	$f_{peak}$ (cycles/mm)	Peak NPS magnitude	NPS AUC
NDCT (Reference)	0.21	1.000	1.000
LDCT (Input)	0.19	3.847	3.612
RED‐CNN	0.14	0.731	0.724
DnCNN	0.15	0.768	0.751
Swin‐Unet	0.18	0.912	0.887
CTformer	0.19	0.953	0.921
MobileMamba‐UNet (Ours)	**0.20**	**0.974**	**0.968**

#### 3.5.4 Computational complexity analysis

To provide a fair and comprehensive evaluation of computational efficiency, we compared all methods under identical conditions using three key indicators: the number of learnable parameters, floating‐point operations per second (GFLOPs), and the average inference time per 512×512 image slice on a single NVIDIA V100 GPU. The results, summarized in Table 8, demonstrate that our proposed MobileMamba‐UNet achieves an excellent balance between model compactness, computational cost, and inference speed.

**TABLE 8:** Computational complexity and inference speed comparison. Best results are in **bold**, second best are underlined.

Method	Params (M)	GFLOPs	Time (ms)
REDCNN	1.856	24.95	32
EDCNN	**0.081**	**1.33**	**4**
Swin‐Unet	29.10	24.13	167
CT‐Mamba	21.28	425.32	563
Ours	5.71	78.8	25

Although our model contains more parameters than the lightweight EDCNN, it significantly reduces the computational burden compared with other recent transformer‐based and SSMs. In particular, MobileMamba‐UNet requires only 78.8 GFLOPs, which is nearly 5.4× lower than CT‐Mamba (425.32 GFLOPs). Furthermore, our model processes each image slice in 25 ms, making it 6.7× faster than Swin‐UNet and over 22× faster than CT‐Mamba, while still maintaining superior reconstruction quality.

This analysis highlights that MobileMamba‐UNet provides an efficient architecture that preserves strong denoising performance with considerably lower computational demand. The model's balance of accuracy, speed, and resource efficiency makes it particularly well‐suited for clinical integration, where rapid and reliable reconstruction is critical for real‐time diagnosis and decision support.

## 4 DISCUSSION

Our experimental results show that MobileMamba‐UNet outperforms both CNN‐based and Transformer‐based methods on the Mayo‐2016 and Mayo‐2020 datasets, achieving higher PSNR and SSIM, along with lower RMSE and LPIPS. These results, supported by visual comparisons, indicate effective noise suppression while preserving anatomical structures and fine details. The MRFFI and WTE‐Mamba modules contribute significantly to multi‐scale feature representation and structural reconstruction, as validated by ablation studies. In addition, MobileMamba‐UNet is computationally efficient, with fewer parameters and lower GFLOPs, enabling fast inference with minimal resource usage. Compared to Swin‐Unet, it is over six times faster while delivering better image quality. This balance of performance and efficiency makes it well‐suited for clinical and resource‐constrained scenarios, highlighting the potential of lightweight architectures with advanced feature fusion for LDCT reconstruction.

However, this study still has several limitations. First, the current model processes 2D slices independently, ignoring inter‐slice information. This may affect the detection of small lesions across slices and the spatial consistency of reconstructed volumes. Although extending the model to 3D could address this issue, it would significantly increase computational cost. A more practical solution is to use a 2.5D strategy, such as stacking adjacent slices or introducing a lightweight inter‐slice attention mechanism. We will explore this in future work. Secondly, patch‐based training limits the receptive field to 256×256, potentially restricting large‐scale context modeling. This effect is alleviated by the WT Enhanced Mamba module, which expands the effective receptive field in the frequency domain, and by full‐resolution (512×512) inference that leverages global context. Nevertheless, training with larger patches or full‐resolution inputs may further improve performance and will be explored in future work. Thirdly, both the Mayo‐2016 and Mayo‐2020 datasets are from Siemens scanners, which may limit generalization to other devices. Applying the model to different scanners may require domain adaptation or fine‐tuning. Evaluating cross‐vendor robustness is also an important direction. Finally, more validation is needed on diverse anatomical regions and ultra‐low‐dose settings. Future work will also consider extending the method to other imaging modalities and conducting clinical studies to further assess its practical value.

## 5 CONCLUSION

In this paper, we proposed MobileMamba‐UNet, a novel architecture for LDCT image reconstruction that effectively reconciles the conflict between high‐fidelity reconstruction and computational efficiency. By seamlessly integrating the MobileMamba backbone into a hierarchical U‐Net structure, our approach overcomes the limitations of existing Transformer‐based methods, which often struggle to balance long‐range dependency modeling with practical memory constraints. Extensive experiments on the Mayo‐2016 and Mayo‐2020 datasets demonstrate that MobileMamba‐UNet achieves state‐of‐the‐art performance across multiple quantitative metrics. Furthermore, the model exhibits significantly lower computational overhead compared to competing approaches, highlighting its potential for efficient deployment in real‐world clinical workflows.

## AUTHOR CONTRIBUTIONS

Jianfang Li conceived and designed the study. Haiyan Liu and Jianfang Li performed the experiments and data analysis. Xiaoli Wang contributed to data curation and result interpretation. Jianshu Hong provided support in maintaining the computing resources and platform.

## CONFLICT OF INTEREST STATEMENT

The authors declare no conflicts of interest.

## Data Availability

The data that support the findings of this study are available from the corresponding author upon reasonable request.
